# Phage-specific antibodies: are they a hurdle for the success of phage therapy?

**DOI:** 10.1042/EBC20240024

**Published:** 2024-12-17

**Authors:** Ayaka Washizaki, Arata Sakiyama, Hiroki Ando

**Affiliations:** 1 Laboratory of Phage Biologics, Graduate School of Medicine, Gifu University, 1-1 Yanagido, Gifu City, Gifu 501-1194, Japan; 2 Center for One Medicine Innovative Translational Research (COMIT), Institute for Advanced Study, Gifu University, 1-1 Yanagido, Gifu City, Gifu 501-1194, Japan; 3 Venture Unit Engineered Phage Therapy, Discovery Accelerator, Astellas Pharma Inc., Tsukuba City, Ibaraki 305-8585, Japan

**Keywords:** antibodies, bacteriophages, phage therapy

## Abstract

Phage therapy has attracted attention again owing to the increasing number of drug-resistant bacteria. Although the efficacy of phage therapy has been reported, numerous studies have indicated that the generation of phage-specific antibodies resulting from phage administration might have an impact on clinical outcomes. Phage-specific antibodies promote phage uptake by macrophages and contribute to their rapid clearance from the body. In addition, phage-specific neutralizing antibodies bind to the phages and diminish their antibacterial activity. Thus, phage-specific antibody production and its role in phage therapy have been analyzed both *in vitro* and *in vivo*. Strategies for prolonging the blood circulation time of phages have also been investigated. However, despite these efforts, the results of clinical trials are still inconsistent, and a consensus on whether phage-specific antibodies influence clinical outcomes has not yet been reached. In this review, we summarize the phage-specific antibody production during phage therapy. In addition, we introduce recently performed clinical trials and discuss whether phage-specific antibodies affect clinical outcomes and what we can do to further improve phage therapy regimens.

## Introduction

Bacteriophages, also known as phages, are viruses that can specifically infect bacteria. From the 1920s to the 1930s, phages were used to treat bacterial infections because they can infect and kill host bacteria. Subsequently, phages were replaced with antibiotics, and phage therapy was forgotten in Western medicine [1]. However, the increasing threat of spreading multidrug-resistant bacteria and sharp decline in antibiotic pipeline have resurrected interest in phage therapy [2]. Although phage therapy has been used for a century, a convincing therapeutic regimen has not yet been established. One of the reasons phage therapy is still under investigation is that, in contrast to antibiotics, the pharmacokinetics (PK) and pharmacodynamics (PD) of phage therapy are difficult to understand [3]. Phage populations can expand in patients following administration if bacterial hosts susceptible to the phage are present in the patient either at the site of infection or elsewhere. At the same time, they are recognized as non-self-antigens and eliminated by the host's immune system or lose activity by neutralizing antibodies. Therefore, the PK and PD can change depending on the replication ability or immunogenicity of the phages used in the study.

The elimination of phages by the immune system has been studied for several decades. Early studies have shown that irradiation of mice delays phage clearance from the liver and spleen [4]. Another study reported that the elimination of coliphage ΦX174 from the blood of immunized mice was significantly faster than non-immunized mice and immunosuppressing agents inhibit the clearance of phages from blood [5]. Moreover, the clearance of coliphage T7 from mouse blood circulation is delayed in B cell-deficient mice. These results strongly suggest that antibodies contribute to the half-life of phages in blood [6].

Antibodies are important factors in determining the PK and PD of phage therapy, and various studies have suggested that antibodies affect the clinical outcomes of phage therapy [7–9]. Therefore, it is important to understand when and which types of phage-specific antibodies are induced after phage administration and how they affect the outcomes of phage therapy. However, information on the relationship between antibodies and clinical outcomes is limited.

In this review, we summarize recent reports on phage-specific antibodies in phage therapy, based on phage-specific antibodies naturally present in humans, antibodies induced during phage therapy, the relationship between phage-specific antibodies and clinical outcomes, and the modification of phages to extend their half-life in the body.

## Do phage-specific antibodies naturally exist in humans?

Phages broadly exist in the environment and are a major component of the human virome [10,11]. This indicates that humans are naturally exposed to phages as antigens. Therefore, we could naturally produce phage-specific antibodies, which we call natural phage-specific antibodies, even if we have never received phage therapy before, and this might affect the speed of phage clearance from the body during phage therapy.

Dąbrowska [12] showed in 2014 that sera from 41 of 50 healthy volunteers had neutralizing activity against coliphage T4 and the neutralizing rate was up to 95%. This shows that healthy individuals produce phage-specific antibodies through natural encounters with phages, which are present and maintained in their serum. Another study showed that 15%, 11%, and 40% of 55 healthy donors had neutralizing activity against *Pseudomonas* phages F8, LMA2, and DP2, respectively, in sera. Although the three phages used in this study were phylogenetically similar, the frequency of individuals with neutralizing activity against each phage was different. Moreover, the study showed that the number of phages that can be neutralized by serum is diverse [13], indicating that the immunogenicity of phages varies from one phage to another, although they are similar. Therefore, if the sera of a patient have neutralizing activity against a few phages, even before phage therapy, we could select similar phages from a phage library that are not recognized by natural phage-specific antibodies.

In contrast, the neutralizing activity was not detected in some cases [14]. Sera from 30 healthy donors and patients infected with *Staphylococcus aureus*, *Pseudomonas aeruginosa*, or *Enterococcus faecalis* showed weak neutralizing activity before phage therapy [15]. In another case, interestingly, although sera from healthy donors showed weak neutralizing activity, patients infected with *S. aureus* showed higher neutralizing activity than that of healthy donors [16,17].

Altogether, these results indicated that phage-specific antibodies were induced by natural exposure to phages. Natural phage-specific antibody titers are higher in individuals with bacterial infections than those without. In addition, the immunogenicity of phages is diverse, and the types of phages neutralized by sera differ from person to person. Therefore, it could be beneficial to confirm the presence of phage-specific antibodies in patient sera before phage therapy and choose phages that are not recognized by natural phage-specific antibodies.

## When and which type of phage-specific antibodies are induced by phage administration?

**Figure 1:**
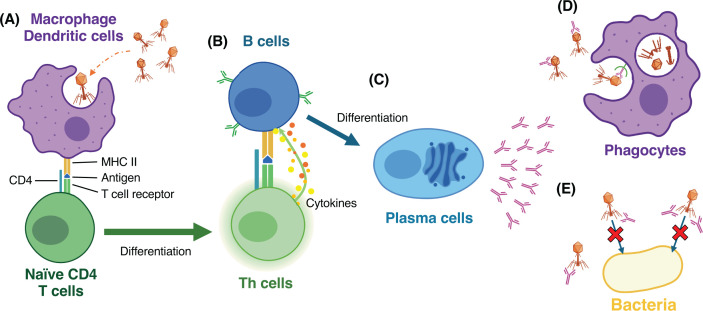
Production of phage-specific antibodies (**A**) Phages are phagocytosed by macrophages or dendritic cells. These cells present a phage protein as an antigen on major histocompatibility complex (MHC) class II molecules and activate naïve T cells. (**B**) T helper (Th) cells interact with MHC class II and T cell receptor. B cells are activated and produce phage-specific antibodies. (**C**) B cells differentiate into plasma cells and secrete phage-specific antibodies. (**D**) Phage-specific antibodies bind to phages and promote phage uptake and degradation by phagocytosis. This leads to rapid clearance of phages from the body. (**E**) Phage-specific antibodies also bind to phages and neutralize antibacterial activity of phages. It results in the reduction of active phage titer.

In phage therapy, patients are injected with an extremely large number of phages compared with that of natural exposure. Therefore, phage-specific antibodies can be induced more extensively, which may affect the clinical outcomes (Figure 1).

**Figure 2:**
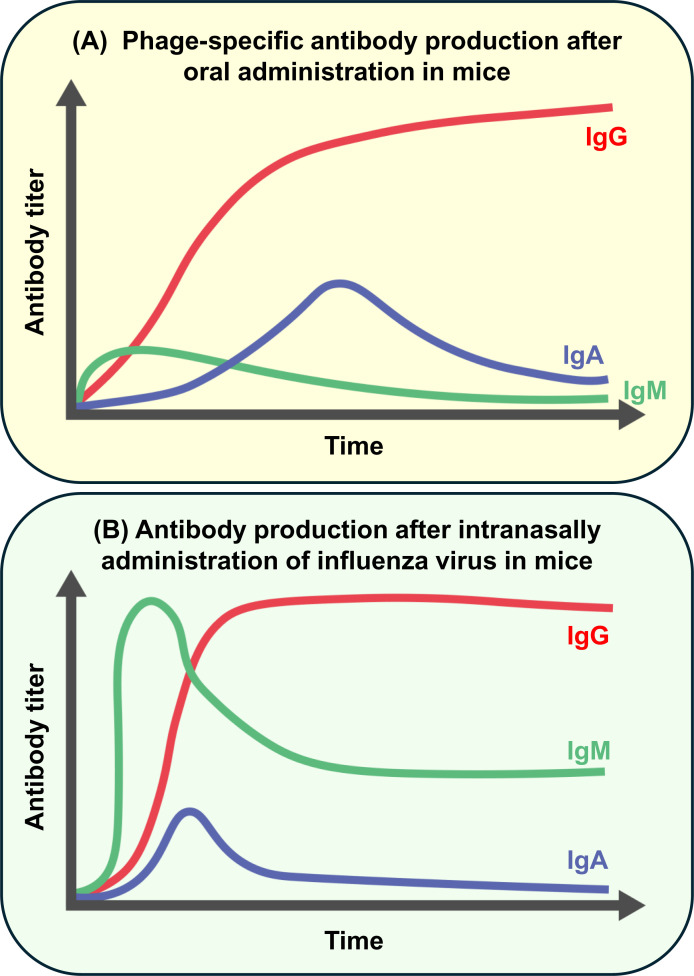
Antibody production after phage or virus infection in mice The order of antibody isotypes produced after phage infection is similar to that after virus infection while the titer or the time of antibody production varies depending on an administration condition. (**A**) Coliphage T4-specific antibody production after oral administration in mice [23]. IgM (green) is weakly produced first. Following IgM, IgG (red) was detected and maintained for a long time. Subsequently, IgA (blue) was produced. (**B**) Antibody production after intranasal virus infection, for example, influenza PR8, in mice [28,29]. IgM (green) is produced immediately after infection, and IgG (red) production follows it. IgA (blue) was produced just after IgG production.

To investigate phage-specific antibody production after phage administration, experiments have been performed using animal models [18–22] (Table 1). In the study by Majewska et al. [23], when mice were orally administered coliphage T4 in drinking water for 100 days, there was no significant increase in immunoglobulin (Ig) M, but IgG in the serum increased from 15 to 36 days. Following IgG production, IgA was induced 64–79 days after the initiation of phage treatment. Importantly, the titer of IgA gradually decreased to a non-significant level once phage administration was interrupted. In contrast to IgA, IgG levels in the blood were maintained even after 100 days of treatment interruption. Moreover, in response to the restart of the second administration of coliphage T4, the IgA titer rapidly recovered. T4 elimination from the blood circulation was more rapid in the seccond administration compared with that of the first administration of T4. Additionally, in this study the authors conducted a similar experiment using subcutaneous injections and found significantly higher titer of phage-specific antibodies (Figure 2A) [23]. Similarly, another experiment using staphylococcal phages showed that IgM levels peaked 10 days after oral administration. In parallel with the decrease in IgM levels, IgG levels increased and plateaued at 50 days. In contrast, the IgA titers increased until two months after phage administration and gradually decreased after the cessation of phage treatment at 120 days. Consistent with the observation in coliphage T4, in the staphylococcal phage, IgA titers immediately increased once the second administration started [24]. In the case of the *Pseudomonas* phage, IgM reached a peak-5–10 days after intraperitoneal (IP) injection. IgG increased following IgM production and the titer was maintained for 60 days [25]. In addition, enterococcal phage injection induced IgM and IgA levels that were 5 and 3800 times, respectively, higher than those present before treatment [26].

**Table 1:** Animal studies detecting phage-specific antibodies after phage administration

Animal	Administration route	Phage	Sample	Antibody isotype	Antibody titer peak time point	Reference
Mice	Orally	T4	Feces	IgA	79 dpi	[23]
			Serum	IgM	15 dpi	
				IgG	36 dpi	
		A3R	Feces	IgA	60 dpi	[24]
			Blood	IgM	21 dpi	
				IgG	50 dpi	
				IgA	60 dpi	
		676Z	Feces	IgA	70 dpi	
			Blood	IgM	21 dpi	
				IgG	50 dpi	
				IgA	60 dpi	
	Intraperitoneally	A3R	Plasma	IgM	10 dpi	[40]
				IgG	30 dpi	
		676Z		IgM	10 dpi	
				IgG	30 dpi	
		F-8	Serum	IgM	10 dpi	[25]
				IgG	20 dpi	
		DSM19872	Plasma	IgM, IgG, IgA	NA*	[18]
		DSM22045				
		536_P3				
		CLB_P2				
		LF110_P3				
		LF73_P1				
		DIJ07_P1				
	Intravenously	PM16	Serum	IgG	7 dpi	[21]
		PM135			7 dpi	
		KP179			7 dpi	
		PA136			7 dpi	
		M13			14 dpi	
Rats	Intravenously	SE_SZW1	Plasma	IgG	NA**	[19]
		AB_SZ6				
Cynomolgus macaques	Intravenously	SE_SZW1	Plasma	IgG		
Calves	Per rectum	φ26	Serum	IgM, IgG, IgA	NA***	[20]
		φ27				
		φ29				
	Intranasally	φ25	Serum	IgM, IgG, IgA		
		A2				
		A5				

dpi, days post infection

NA, not analyzed

*Antibody titer peak time point was unknown because data was measured at 10 and 25 dpi only.

**Antibody was checked by western blot but titer was not quantified.

***Antibody titer peak time point was unknown because data was measured at 5 dpi only.

Antibody production after phage infection is similar to conventional virus infection (Figure 2). For example, when influenza A virus A/Puerto Rico/8 (influenza PR8) was intranasally inoculated in mice, IgM was first produced. Subsequently, a class switch from IgM to IgG occurred; finally, IgA was induced (Figure 2B) [9,27–29]. Though the titer or timing of each antibody isotype is slightly different depending on phage immunogenicity, administration route, or dose, phages may be recognized similarly to conventional viruses.

## Administration route and phage-specific antibody induction

Previous studies involving only phage without bacterial infection have shown that the distribution of phages in the mouse body differs according to the route of administration. Geier et al. [30] tested phage distribution in the mouse body after IP, intramuscular (IM), intravenous (IV), and oral administration. After oral administration, phages were recovered from the liver and spleen; however, the percentage of phages recovered from each organ was lower than that of the injected phage. In contrast, in other administration routes, especially IP or IV injection, phages were detected in various organs, and the highest plaque-forming units (PFUs) were observed in the spleen, which plays a major role in antibody production. These studies suggest that phage distribution in the body differs depending on the route of administration, which could affect antibody induction [31,32].

Oral administration tends to produce weak phage-specific antibodies. Bruttin and Brüssow [14] orally administered coliphage T4 twice in humans; however, phage-specific antibodies were not detected at the end of the experiment. This suggests that longitudinal oral administration is required to produce phage-specific antibodies. This is supported by the finding that it took more than 15 days to start IgG induction by oral administration of T4 to mice [23]. Interestingly, although 2 weeks of oral phage administration in drinking water failed to induce phage-specific antibodies, one shot of IP phage injection provided high neutralizing activity to sera [33]. Moreover, one shot of ΦX174 IV injection caused phage-specific antibody induction and the second shot at day 42 boosted phage-specific antibody titer [34].

In the case of nebulized phage therapy against *S. aureus* or *P. aeruginosa*, a recent report showed that plasma acquired phage-neutralizing activity after 10 days treatment. However, the kinetics of phage-specific antibody production could not be studied because patients had phage-specific antibodies before the therapy started. However, this study suggests that nebulization also induces phage-specific antibodies [35].

To choose an appropriate administration route, in addition to phage-specific antibody induction, phage delivery to an infection site is also an important factor [36]. For example, oral administration generally showed low inducibility of phage-specific antibodies, but the acidic environment of the stomach caused phage deactivation. IP and IV injections tended to induce a higher titer of phage-specific antibodies; however, phage is immediately distributed to the whole body. Therefore, it would be appropriate for infection after transplantation or bacteremia. Inhalation administration, such as nebulization can efficiently deliver phage to lung and is used in therapy for cystic fibrosis or pulmonary infection. To achieve success in therapy, we need to carefully consider a balance between efficient phage delivery to an infection site and phage-specific antibody production, and choose an appropriate administration route and schedule.

## Which phage proteins can be an antigen or a target for neutralization?

Phages are complex structures comprising many proteins. Therefore, phage-specific antibodies against various phage proteins can be induced. Previous studies have suggested that many structural proteins function as immunogens during humoral immune responses to phages [12,24,26,37–39].

In a study that investigated the immunogenicity of structural proteins of coliphage T4, mice were treated with the phage orally for 100 days, and IgG or IgA antibodies specific for head proteins (gp23, gp24, Hoc, and Soc) or short-tail fiber protein (gp12) were analyzed. The titers of antibodies specific to Hoc and gp12 significantly increased, indicating that these were highly immunogenic among the tested proteins [23]. Another study showed that IP injection of T4 induced a significant number of Hoc-specific antibodies [12]. Majewska et al. [24] analyzed the induction of major capsid protein (Mcp), tail morphogenic protein (TmpH), and base plate protein H (gpORF096)-specific antibodies after oral administration of staphylococcal phages. The authors observed a production of antibodies specific for Mcp and TmpH, which exist at high copy numbers in virions. However, antibodies against gpORF096, which are less abundant in virions, did not induce a significant response. These results suggest that the copy number of structural proteins in a virion may contribute to immunogenicity.

Although various phage-specific antibodies are induced by phage therapy, not all antibodies exhibit phage-neutralizing activity. For example, in *Pseudomonas* phages, the titer of head-specific antibodies is not significantly different between neutralization-negative and neutralization-positive sera [13]. This suggests that head-specific antibodies do not contribute to phage neutralization. In addition, mice were immunized with head proteins (Mcp and gpORF059), tail proteins (TmpH), or baseplates (pgORF096) of the staphylococcal phages. However, phage-neutralizing activity has been observed only in the sera of gpORF096-immunized mice [40]. Therefore, antibodies specific for a region involved in phage-bacteria interactions could work as neutralizing antibodies.

## Do phage-specific antibodies influence the outcome of phage therapy?

Although various clinical trials of phage therapy have been reported, with a mixture of successful and failed cases, and an appropriate regimen that leads to treatment success is still being investigated [41,42]. In this context, the relationship between the induction of phage-specific antibodies and the results of therapy has been investigated in animal models and clinical trials [43–45].

In the mouse models, the efficacy of treatment was assessed with or without phage immunization before bacterial infection. When mice were immunized with phages before treatment, phage clearance from the body was significantly faster than in mice administered the vehicle control. In addition, phage therapy did not reduce the number of colony-forming units (CFUs) in feces when mice were immunized before treatment [46]. Another study showed that pre-immunization with phage delayed the reduction of wound size or complete closure of the wound in the *Acinetobacter baumannii* mouse wound infection model [47]. These results suggest that phage-specific antibodies have an impact on the efficacy of therapy. However, observation from clinical trials are controversial.

In phage therapy against *Mycobacterium abscessus* infection, Dedrick et al. [48] reported that the CFU in sputum decreased once. However, 2 months after phage therapy started, CFU in sputum rebounded in parallel with the production of neutralizing antibodies. Eventually, the clinical trial failed to eliminate bacteria, suggesting that neutralizing antibodies might hamper the killing of bacteria by phages, leading to clinical failure. In a different study, patients were treated with the same phage cocktail. At that time, the patients were immunosuppressed after the lung transplantation. Therefore, neutralizing antibodies were not induced and a successful result was obtained [37]. Similarly, phage therapy against *P. aeruginosa* was successful in a toddler after liver transplantation, without antibody-mediated phage neutralization [49]. When antibiotics were used together with *Mycobacterium chelonae* phages, phage-specific antibodies were detected at 17 days and were maintained for 16 weeks. The neutralizing activity in the sera also started to increase at day 17, and the augmentation continued until week 16. However, clinical success was achieved in this trial [50]. It has been suggested that *M. chelonae* phages dramatically decrease bacteria at a very early period of therapy, making it a good environment for antibiotics to work efficiently.

Dedrick et al. [51] reported phage therapy for mycobacterial infection in 20 patients, including three cases shown in the previous paragraph. In this report, the neutralizing activity of sera from 15 of 20 patients was analyzed and robust neutralization was observed in nine patients. However, the correlation between neutralization and outcome was not detected. In Pirnay et al.’s study [52] showing outcomes for 100 cases of personalized phage therapy, phage neutralization in sera was tested in 13 patients. Although sera from 5 of 13 patients neutralized phage, 4 of these 5 patients achieved eradication of target bacteria. Another report showed that some patients had a significant increase in phage-specific antibodies and the titer of phage-specific antibodies was correlated with the neutralizing activity of sera. However, no correlation has been observed between the neutralization activity of sera and the clinical outcomes [15–17].

These studies have shown controversial results, and the relationship between phage-specific antibodies and clinical outcomes remains unclear. However, the effect of phage-specific antibodies can be minimized by choosing an appropriate administration route, dose, or a phage with low immunogenicity. In addition, a combination of antibiotics and phage therapy may be effective, even in the presence of phage-specific antibodies.

## Can we protect and prolong circulation time of phages in the human body?

**Figure 3:**
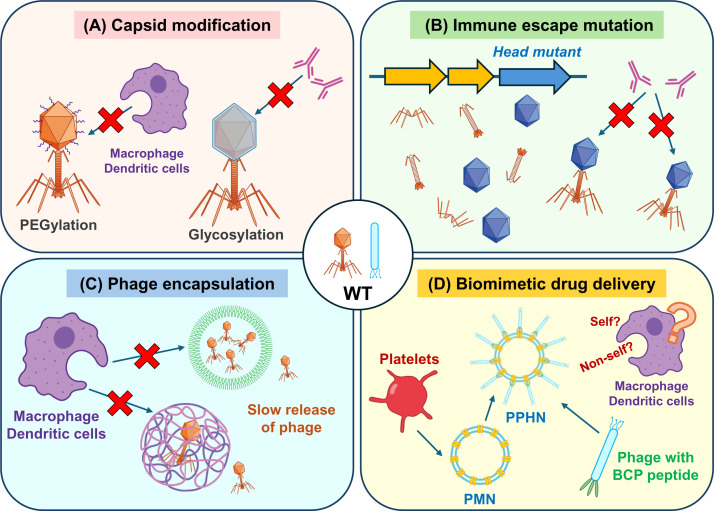
Strategies to protect and prolong circulation time of phages in human body (**A**) Capsid modifications such as glycosylation works as shield against phage-specific antibodies. PEGylation of phage can prolong circulation time in human body. (**B**) Engineered phages harboring an immune-escape mutation(s). These engineered phages could avoid recognition by immune system. (**C**) Phage encapsulations enable slow release of phages. It results in low phage-specific antibody production and long retention of phage in blood. (**D**) In biomimetic drug delivery, platelet membrane nanoparticles (PMNs) are prepared from platelets. At the same time, engineered phages with blood circulation-prolonging (BCP) peptide are prepared. Then phage-platelet hybrid nanoparticle (PPHN) is composed from PMN and the engineered phage. This system shows long circulation of phage and low immunogenicity.

The rapid clearance of phages from bodies is caused by phage-specific antibodies, and it is a major concern. Therefore, efforts have been made to overcome this issue by employing appropriate drug delivery systems or the modification of phages or both [53,54] (Figure 3).

Kim et al. [55] showed that conjugation of polyethylene glycol (PEGylation) to *Salmonella* phages prolonged blood circulation time in non-immunized animals (Figure 3A). A recent study showed that specific types of mycobacterial phages have a glycosylase gene and that the capsid or tail or both are O-glycosylated. Glycosylation worked as a shield and prevented binding of phage-specific antibodies, and phage neutralization by sera was weaker than that of the non-glycosylated mutant phage [56] (Figure 3A).

Mutant phages of coliphage λ and *Salmonella* phage P22, which can circulate in the blood for a longer time than the wild-type, were isolated by serial passage. These phages have a mutation in the head protein, which causes a change in circulation time in the blood [57,58] (Figure 3B).

Unique delivery systems have been used for phage delivery. Kim et al. [54] employed polylactic-co-glycolic acid/alginate-composite encapsulation of phages. Phages were slowly released from the capsules, and PFUs were detectable in the spleen even 28 days post-administration (Figure 3C). Moreover, the induction of phage-specific antibodies was weaker.

Knowledge from studies using phage display could apply to phage therapy. For example, Jin et al. [59] found that a blood circulation-prolonging (BCP) peptide prolonged the blood circulation time of doxorubicin-loaded human ferritin nanocages using phage display. They applied this method to phage therapy and constructed coliphage M13 harboring BCP peptide and BglII, which is a restriction endonuclease from *Bacillus globigii*, to enhance antibacterial activity. Although this engineered phage showed a longer blood circulation time than the original phage, the engineered phage was further combined with platelet membrane nanoparticles to prepare phage-platelet hybrid nanoparticles. This system showed an additional extension of the half-life of phages while maintaining their antibacterial activity [60] (Figure 3D). Peptides that change biodistribution or homing of phage were found by using phage display [61]. These peptides might apply to phage therapy and enable phage to preferentially distribute to a target organ and avoid accumulation in secondary lymphoid organs.

## Conclusion remarks

In this review, we summarize the recent knowledge about phage-specific antibodies produced in phage therapy. Phages are non-self-antigens that stimulate the T-cell-dependent immune system. Phage-specific antibodies contribute to the rapid elimination of phages from the blood circulation or diminish their killing activity.

When phages are administered to animal models, phage-specific antibodies are produced in the following order: IgM, IgG, and IgA. Sera containing phage-specific neutralizing antibodies can reduce PFU of phage by binding to phages. In addition, even phage-specific non-neutralizing antibodies can play a role in the rapid degradation of phages by opsonization and promotion of phagocytosis by macrophages [62–64]. Administration routes play a significant role in antibody induction. IP and IV injections tended to result in much stronger antibody production than oral or topical administration. Although this is a general tendency in the relationship between the administration route and phage-specific antibody induction, the titer of phage-specific antibodies produced during therapy also depends on other factors, such as the immunogenicity of each phage, immune status of the patient, dose of phage, and administration schedule.

To date, there is no consensus regarding whether phage-specific antibodies affect the clinical outcomes of phage therapy. However, efforts to find a strategy to extend the phage circulation time are worthwhile. From this perspective, encapsulation, biomimetic drug delivery systems, and modification of phage protein may be promising methods. Phage engineering is one of the hot topics in phage therapy and researchers have been developing approaches such as recombineering, CRISPR-Cas system, yeast-based genome assembly, and synthetic engineering [65–67]. Engineered phages can compensate for limitations such as narrow host specificity and the risk of lysogeny [65,68]. Engineering enables phages to possess additional bactericidal abilities [69,70] and various phage engineering methods have been developed. Therefore, if immune escape mutations are embedded in phages using engineering technology, the phages could more effectively reduce the number of target bacteria.

Phage therapy has a history of more than 100 years; nevertheless, the factors that determine therapeutic outcomes are complicated, and a robust therapeutic strategy has not been established. Regarding phage-specific antibodies, information on the types of antibodies induced during therapy or the relationship between the administration route and phage-specific antibodies has accumulated. However, knowledge about the immunogenicity of each phage or the modification or mutation of phages to escape from the immune system is insufficient. Further studies should provide consensus criteria to conduct an optimized phage therapy for each patient based on factors such as antibiotic sensitivity of bacteria, disease status, immune status of the patient, and immunogenicity of the phage.

## Summary

- Phage-specific antibodies are induced during phage therapy and affect PK and PD.
- The induction of phage-specific antibodies is diverse owing to many factors, such as the type of phage, immune state of a patient, administration route, dose, and administration schedule.
- Rapid clearance or neutralization of phages by phage-specific antibodies may affect therapeutic efficacy.
- Strategies for prolonging the half-life of phages could enhance efficacy of phage therapy.

## Abbreviations

BCP: blood circulation-prolonging
CFU: colony-forming unit
Ig: immunoglobulin
IM: intramuscular
influenza PR8: influenza A virus A/Puerto Rico/8
IP: intraperitoneal
IV: intravenous
Mcp: major capsid protein
MHC: major histocompatibility complex
PD: pharmacodynamics
PFU: plaque-forming unit
pgORF096: base plate protein H
PK: pharmacokinetics
PMN: platelet membrane nanoparticle
PPHN: phage-platelet hybrid nanoparticle
Th: T helper
TmpH: tail morphogenic protein
